# Perioperative Protein and Vitamin Supplementation in Plastic Surgery: An Evidence-Based Proposal for an Assessment and Replacement Protocol

**DOI:** 10.7759/cureus.111477

**Published:** 2026-06-25

**Authors:** Luiz Augusto Sousa Oliveira, Nélio Nunes Cabette Filho, Victor Da Costa Sacksida Valladão, Ligia H Mendes, Fábio André Zanella

**Affiliations:** 1 General Surgery, Fundação Hospitalar Santa Terezinha de Erechim (FHSTE), Erechim, BRA; 2 Plastic Surgery, Hospital Niterói D'Or, Serviço de Pós-Graduação em Cirurgia Plástica Prof. Ronaldo Pontes, Niterói, BRA; 3 Plastic Surgery, Hospital Nossa Senhora da Conceição, Grupo Hospitalar Conceição, Porto Alegre, BRA

**Keywords:** dietary proteins, enhanced recovery after surgery, nutritional support, perioperative care, plastic surgery, wound healing

## Abstract

Wound complications, dehiscence, and surgical-site infection remain relevant determinants of aesthetic and functional outcomes in plastic surgery and appear to bear a measurable relationship with the patient’s perioperative nutritional status. The progressive incorporation of Enhanced Recovery After Surgery (ERAS) protocols into plastic surgery, particularly in autologous breast reconstruction and post-bariatric body contouring, has increasingly positioned preoperative nutritional optimization as a structural component of care rather than an optional adjunct. This narrative review consolidates current evidence on perioperative protein and vitamin supplementation (covering a window of approximately seven to fourteen days before and after surgery) in plastic surgery, with a focus on American and European literature and on consensus guidelines from the ERAS Society and European Society for Clinical Nutrition and Metabolism. Hypoalbuminemia emerges as one of the more consistently reported predictors of postoperative morbidity in plastic surgery National Surgical Quality Improvement Program analyses, although the strength of this association is not uniform across studies; immunonutrition with arginine and omega-3 fatty acids has relatively robust support in major surgery and has already been incorporated into ERAS pathways for breast reconstruction. Patients undergoing post-bariatric body contouring appear to represent a higher-risk population, with a reported high prevalence of iron, vitamin B12, vitamin D, zinc, and protein deficiencies. From this evidence base, we propose a structured protocol combining preoperative laboratory screening, risk stratification, and targeted replacement of documented deficiencies, accompanied by oral protein supplementation and, in selected cases, immunonutrition. The protocol aims to balance clinical applicability and methodological caution, restricting recommendations to interventions with reasonably consistent evidentiary support.

## Introduction and background

Surgical wound complications, including dehiscence, partial flap necrosis, surgical-site infection, and delayed healing, remain among the principal modifiable determinants of aesthetic and functional outcomes in plastic surgery. Even when limited in extent, such events can substantially compromise the final result of procedures whose primary endpoint is morphological precision [1,2]. Among the modifiable preoperative variables, the patient’s nutritional status occupies a particularly relevant place, as it can influence collagen synthesis, immune competence, and the metabolic capacity to cope with the catabolic stress of surgery [3,4].

Over the past decade, the incorporation of Enhanced Recovery After Surgery (ERAS) protocols, multimodal, evidence-based perioperative care pathways designed to attenuate the surgical stress response and accelerate functional recovery, into plastic surgery has progressively reorganized perioperative care according to standardized bundles [5,6]. The recommendations of the ERAS Society for breast reconstruction [5] and their subsequent expansion to body contouring [7,8] have helped position preoperative nutritional optimization, abandonment of prolonged fasting, and preoperative carbohydrate loading as increasingly common components of contemporary practice. Despite this trend, nutritional intervention is still applied inconsistently in routine plastic surgery practice, with frequent reliance on heterogeneous and poorly individualized recommendations.

This article aims to consolidate the current evidence on perioperative protein and vitamin supplementation in plastic surgery, focusing on the window of approximately seven to fourteen days before and after the procedure, and to propose a structured protocol for preoperative assessment and targeted replacement, grounded in the most robust evidence available in American and European literature.

## Review

Methodology

This is a structured narrative review. Searches were conducted in PubMed, Embase, and the Cochrane Library, covering the period from January 2015 to March 2026, with selective inclusion of seminal references published earlier when they remain currently cited and clinically authoritative (notably ERAS Society and ESPEN consensus documents). The search strings combined the terms “perioperative nutrition,” “protein supplementation,” “immunonutrition,” “micronutrients,” “wound healing,” “ERAS,” “plastic surgery,” “breast reconstruction,” and “body contouring.”

Priority was given to randomized controlled trials, meta-analyses, large National Surgical Quality Improvement Program (NSQIP)-based or multicenter cohort studies, and formal guidelines from the ERAS Society and European Society for Clinical Nutrition and Metabolism (ESPEN). Case reports, studies with fewer than 30 patients, and opinion articles without supporting data were excluded. Selection privileged consistency of evidence in plastic surgery populations or its defensible extrapolation from major elective surgery. Topics with weak or contradictory evidence in plastic surgery were explicitly excluded from the protocol recommendations and listed as out-of-scope at the end of the article.

Physiological basis and the perioperative window

Surgery typically induces a catabolic response characterized by negative nitrogen balance, insulin resistance, and increased systemic inflammation, whose magnitude tends to parallel the extent of the procedure and the patient’s preoperative metabolic reserve [3,9]. Wound healing comprises sequential phases, i.e., inflammatory, proliferative, and remodeling, each with specific demands for amino acid availability, micronutrient cofactors, and adequate tissue oxygenation [4,10]. Inadequate substrate supply at any of these stages may compromise collagen synthesis, neoangiogenesis, and the immune response, with a potential clinical impact on the rate of wound complications [11,12].

The 7-14-day window before and after surgery is often regarded as the period in which targeted nutritional interventions may offer the most favorable cost-effectiveness ratio. Preoperatively, this window may be sufficient to attenuate insulin resistance, optimize visceral protein stores, and prepare the immune response [5,9]. Postoperatively, it covers the critical phase of catabolism and the beginning of the proliferative phase of healing, when protein and energy demands tend to be highest [3,12].

Protein and global nutritional status

Preoperative hypoalbuminemia is among the most consistently studied nutritional markers associated with morbidity in plastic surgery. In several large NSQIP-based analyses of immediate breast reconstruction, abdominoplasty, and post-bariatric body contouring, serum albumin below 3.5 g/dL has been independently associated with higher rates of surgical-site infection, dehiscence, reoperation, and prolonged length of stay [11,13,14]. It should be noted, however, that this association is not entirely uniform across the literature, and its apparent strength may vary with the procedure, the patient population, and concurrent risk factors; albumin is therefore best interpreted within the broader clinical context rather than as an isolated determinant. Moreover, because albumin is a negative acute-phase reactant, its serum concentration falls in response to systemic inflammation independently of nutritional intake. Therefore, preoperative hypoalbuminemia reflects inflammatory burden as much as protein malnutrition, which further argues against its interpretation as a stand-alone nutritional marker. Pre-albumin (transthyretin), with a shorter half-life, may offer complementary value when available, particularly in patients with recent weight loss or under medical optimization [15].

The ESPEN guidelines on perioperative clinical nutrition recommend a protein intake of 1.2 to 1.5 g/kg/day in surgical patients, with values up to 2.0 g/kg/day in catabolic conditions or major weight loss [9,16]. Oral supplementation with whey-protein-based formulas, given their rapid digestion and high leucine content, represents a practical and generally well-tolerated strategy for restoring this intake in the preoperative window [17,18]. Among elderly or sarcopenic patients, evidence from major elective surgery supports, where feasible, continuation of supplementation for at least seven to fourteen days postoperatively to help attenuate lean-mass loss and preserve healing capacity [17,19].

Immunonutrition

Immunonutrition formulations, typically combining arginine, omega-3 polyunsaturated fatty acids, and nucleotides, represent one of the better-studied nutritional interventions in major elective surgery. Meta-analyses by Drover et al. (2011) [20] and Marimuthu et al. (2012) [21] reported reductions in infectious complications and length of stay in gastrointestinal and oncological surgery, with the most consistent effect generally observed when supplementation is initiated five to seven days preoperatively. A commonly described regimen consists of three daily servings of an immunonutrient formula for five to seven days before surgery, with optional continuation for up to seven days postoperatively in cases of greater catabolic load [20,21].

In plastic surgery, the most developed evidence base is in autologous breast reconstruction. The ERAS Society consensus for breast reconstruction (2017) includes immunonutrition among its formal recommendations [5], supported by single-center experiences suggesting reductions in morbidity and length of stay in deep inferior epigastric perforator and other free-flap reconstructions [7,22]. Although procedure-specific randomized controlled trials in plastic surgery remain scarce, extrapolation from major surgery appears reasonable for procedures of comparable magnitude, namely, extensive body contouring, autologous reconstruction, and complex secondary procedures.

Micronutrients with consistent evidence in surgical wound healing

Among the micronutrients, those with comparatively more consistent evidence in the context of surgical wound healing are vitamin C, vitamin A, zinc, and the hematinics (iron, vitamin B12, folate). Vitamin C is an essential cofactor in collagen hydroxylation; clinically relevant deficiency, more frequent in elderly patients, smokers, and post-bariatric patients, has been associated with impaired wound healing [4,23]. Vitamin A appears to counteract the inhibitory effect of corticosteroids on healing and may be clinically relevant in patients on chronic immunosuppression [24]. Zinc participates in DNA synthesis and epithelialization; its deficiency, frequently documented in post-bariatric body contouring candidates, may justify screening in this group [25,26].

The hematinics deserve particular emphasis. Preoperative anemia has been consistently associated with increased perioperative morbidity, prolonged hospital stay, and higher rates of allogeneic transfusion in major surgery, with impact also reported in plastic surgery cohorts [27,28]. Iron deficiency, with or without anemia, together with vitamin B12 and folate deficiencies, shows a high prevalence in post-bariatric patients and in those subjected to chronic restrictive diets [26,29]. Their correction in the preoperative window may improve tissue oxygenation and reduce transfusion needs, with potential implications for the outcome of microsurgical and reconstructive procedures [27].

High-risk populations in plastic surgery

The post-bariatric body contouring patient is perhaps the clearest example of a population in which targeted nutritional intervention appears justified. Studies by Agha-Mohammadi and Hurwitz [26] and by Naghshineh et al. [29] reported prevalences exceeding 50% for at least one micronutrient deficiency among candidates for contouring procedures, with frequent involvement of iron, vitamin B12, vitamin D, zinc, and protein. Such deficiencies have been associated with higher rates of wound complications and dehiscence after extensive procedures such as fleur-de-lis abdominoplasty, body lift, and combined contouring [26,29,30].

Other groups that may warrant structured nutritional screening include elderly and sarcopenic patients, in whom preoperative protein reserve may modulate the risk of complications and the recovery of functional capacity [19,31]; patients with type 2 diabetes mellitus and chronic smokers, in whom impaired tissue oxygenation may amplify the impact of any micronutrient deficiency [12,30]; patients on chronic restrictive diets, including strict vegans, with relevant risk for vitamin B12, iron, and zinc deficiencies [29]; and oncological patients undergoing reconstruction, in whom underlying disease and adjuvant therapies frequently compound the nutritional risk [3,5].

Preoperative fasting and carbohydrate loading

The abandonment of prolonged preoperative fasting is one of the more established elements of contemporary ERAS protocols. ESPEN and ERAS Society recommendations support intake of clear fluids up to two hours before surgery and the use of preoperative carbohydrate loading in the form of maltodextrin-based beverages [5,9,32]. In elective plastic surgery, carbohydrate loading may reduce insulin resistance, attenuate the postoperative catabolic response, and decrease the subjective discomfort associated with prolonged fasting, with a generally favorable safety profile when administered to non-diabetic patients without significant gastroparesis [32].

Proposed protocol

**Figure 1:**
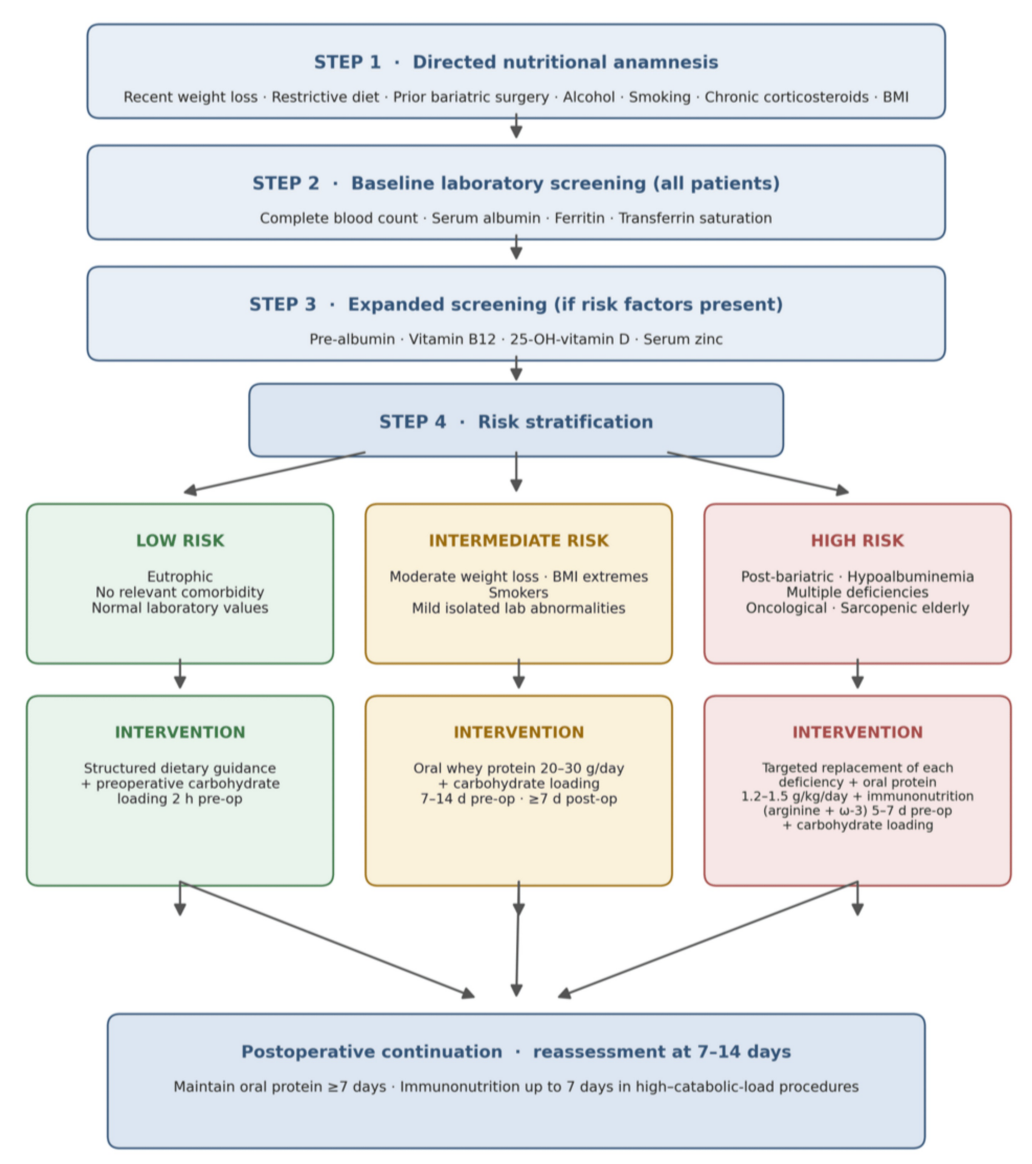
Proposed algorithm for perioperative nutritional assessment and intervention in elective plastic surgery. The algorithm proceeds through directed nutritional anamnesis (step 1); baseline laboratory screening for all patients (step 2); expanded screening when risk factors are present (step 3); risk stratification into low, intermediate, and high categories (step 4); targeted intervention (step 5); and postoperative continuation with reassessment at 7–14 days (step 6). Adapted from ERAS Society (Temple-Oberle et al., 2017) [5] and ESPEN (Weimann et al., 2021) [9]. BMI: body mass index; 25-OH-vitamin D: 25-hydroxyvitamin D; ω-3: omega-3 polyunsaturated fatty acids; ERAS: Enhanced Recovery After Surgery

From this evidence base, we propose a structured perioperative nutritional protocol applicable to elective plastic surgery, organized in the following three components: assessment, risk stratification, and targeted intervention (Figure 1).

Preoperative assessment should include directed nutritional anamnesis (recent weight loss, restrictive diets, prior bariatric surgery, alcohol consumption, smoking, chronic corticosteroid use), measurement of body mass index, and laboratory screening. The baseline laboratory panel comprises complete blood count, serum albumin, and ferritin with transferrin saturation. In patients at higher risk, i.e., post-bariatric, advanced age, body mass index (BMI) extremes, restrictive diets, oncological, the panel is expanded to include pre-albumin, vitamin B12, 25-hydroxyvitamin D, and serum zinc [5,9,26,29].

Risk stratification distinguishes three categories. Low-risk patients (eutrophic, no relevant comorbidities, normal laboratory values) receive structured dietary guidance and preoperative carbohydrate loading. Intermediate-risk patients (moderate weight loss, BMI in extremes, smokers, isolated mild laboratory abnormalities) receive, in addition, oral protein supplementation of 20 to 30 g/day during the seven to fourteen days preceding surgery, with continuation for at least seven days postoperatively. High-risk patients (post-bariatric, marked hypoalbuminemia, multiple documented deficiencies, oncological reconstruction, sarcopenic elderly) receive expanded screening, targeted replacement of each identified deficiency, oral protein supplementation, and immunonutrition for five to seven days preoperatively, with continuation for up to seven days postoperatively in procedures of higher catabolic load [5,9,20,21,26,29]. Table 1 summarizes the recommendations by category.

**Table 1:** Perioperative nutritional recommendations by risk category in elective plastic surgery. Recommendations adapted from ERAS Society (Temple-Oberle et al., 2017) [5] and ESPEN (Weimann et al., 2021) [9]. CBC: complete blood count; BMI: body mass index; 25-OH-vitamin D: 25-hydroxyvitamin D; ω-3: omega-3 polyunsaturated fatty acids; d: days; h: hours

Risk category	Profile	Laboratory screening	Nutritional intervention	Window
Low	Eutrophic, no relevant comorbidity, normal labs	CBC, serum albumin, ferritin, transferrin saturation	Structured dietary guidance; preoperative carbohydrate loading 2 hours preoperatively	Carbohydrate loading 2 hours preoperatively
Intermediate	Moderate weight loss, BMI extremes, smokers, mild isolated lab abnormalities	Baseline panel + repeat at 2 weeks if abnormal	Oral whey protein 20–30 g/day, carbohydrate loading	7–14 days preoperatively; ≥7 days postoperatively
High	Post-bariatric; marked hypoalbuminemia, multiple deficiencies, oncological reconstruction, sarcopenic elderly	Baseline panel + pre-albumin, vitamin B12, 25-OH-vitamin D, serum zinc	Targeted replacement of each deficiency + oral protein 1.2–1.5 g/kg/day + immunonutrition (arginine + ω-3) 3×/day for 5–7 days preoperatively; carbohydrate loading	5–7 days preoperative immunonutrition; protein 7–14 days preoperatively and up to 7 days postoperatively

Discussion

The body of evidence supporting structured perioperative nutritional intervention in plastic surgery has grown substantially over the past decade. Hypoalbuminemia as a marker associated with complications [11,13,14], immunonutrition in major surgery and breast reconstruction [5,20,21,22], the high prevalence of micronutrient deficiencies in post-bariatric candidates [26,29], and the abandonment of prolonged fasting in favor of carbohydrate loading [9,32] together provide a reasonably consistent foundation on which a clinically applicable protocol can be built.

Beyond confirming the direction of the evidence, the principal contribution of this review is to translate it into an operational sequence. The three components of the proposed protocol map directly onto the six steps of the algorithm (Figure 1): assessment corresponds to directed nutritional anamnesis and baseline laboratory screening (steps 1-2) [5,9]; risk stratification corresponds to expanded screening when risk factors are present and to allocation into low-, intermediate-, and high-risk categories (steps 3-4), informed by the elevated deficiency prevalence documented in post-bariatric and other high-risk candidates [26,29]; and targeted intervention corresponds to category-specific replacement, oral protein supplementation, and immunonutrition in selected cases, with postoperative continuation and reassessment at 7-14 days (steps 5-6) [9,16,20,21]. This structure is intended to make the protocol reproducible across services with differing resources: the baseline panel (complete blood count, albumin, and ferritin with transferrin saturation) is inexpensive and widely available, whereas the expanded panel and immunonutrition are reserved for the patients most likely to benefit, limiting cost and complexity. Framing the recommendations as a stepwise decision pathway rather than a uniform prescription also helps avoid the empirical universal replacement that current evidence does not support.

Limitations

The principal limitation of this body of evidence is the relative scarcity of randomized controlled trials specifically designed for plastic surgery populations. Much of the available evidence derives from major general, oncological, and bariatric surgery, with extrapolation supported by physiological plausibility and analogous risk profile. Additionally, heterogeneity of supplementation formulations, doses, and duration of intervention hinders objective quantitative synthesis. Future prospective studies in well-defined plastic surgery cohorts, particularly post-bariatric body contouring, autologous breast reconstruction, and complex secondary procedures, would substantially consolidate the field.

Topics excluded due to weak evidence in plastic surgery

The following interventions were deliberately excluded from the protocol recommendations because current evidence in plastic surgery is weak, inconsistent, or absent: (1) Routine vitamin D supplementation isolated for plastic surgery outcomes (inconsistent evidence). (2) Routine selenium, copper, and manganese supplementation (no procedure-specific evidence). (3) Isolated β-hydroxy-β-methylbutyrate outside sarcopenic contexts. (4) Commercial antioxidant complexes without controlled trials. (5) Curcumin, bromelain, and arnica (related to ecchymosis/edema control, not structural healing). (6) Probiotics for plastic surgery wound outcomes (evidence still preliminary). (7) Universal empirical replacement regardless of risk stratification.

## Conclusions

Current evidence supports perioperative nutritional optimization as an increasingly important component of care in plastic surgery, with a plausible impact on wound complications and recovery quality. Preoperative hypoalbuminemia, micronutrient deficiencies in post-bariatric patients, and the benefit of immunonutrition in major surgery are reasonably well-established and provide a useful basis for a structured protocol. The proposed protocol, namely, assessment, risk stratification, and targeted replacement, seeks to translate this evidence into clinical applicability while restricting recommendations to interventions with reasonably consistent evidentiary support. Empirical universal replacement does not appear justified and was not included. The incorporation of structured nutritional screening into plastic surgery practice may represent a practical opportunity to reduce morbidity and improve outcome predictability.
